# Real and Imagined Smellscapes

**DOI:** 10.3389/fpsyg.2021.718172

**Published:** 2021-12-24

**Authors:** PerMagnus Lindborg, Kongmeng Liew

**Affiliations:** ^1^SoundLab, School of Creative Media, City University of Hong Kong, Kowloon, Hong Kong SAR, China; ^2^Social Computing Laboratory, Division of Information Sciences, Nara Institute of Science and Technology, Ikoma, Japan

**Keywords:** smellscape, environment, perception, smell, imagination, memory, crossmodal

## Abstract

The smellscape is the olfactory environment as perceived and understood, consisting of odours and scents from multiple smell sources. To what extent can audiovisual information evoke the smells of a real, complex, and multimodal environment? To investigate smellscape imagination, we compared results from two studies. In the first, onsite participants (*N* = 15) made a sensory walk through seven locations of an open-air market. In the second, online participants (*N* = 53) made a virtual walk through the same locations reproduced with audio and video recordings. Responses in the form of free-form verbal annotations, ratings with semantic scales, and a ‘smell wheel’, were analysed for environmental quality, smell source type and strength, and hedonic tone. The degree of association between real and imagined smellscapes was measured through canonical correlation analysis. Hedonic tone, as expressed through frequency counts of keywords in free-form annotations was significantly associated, suggesting that smell sources might generally be correctly inferred from audiovisual information, when such imagination is required. On the other hand, onsite ratings of olfactory quality were not significantly associated with online ratings of audiovisual reproductions, when participants were not specifically asked to imagine smells. We discuss findings in the light of cross-modal association, categorisation, and memory recall of smells.

## Introduction

The term ‘smellscape’ was introduced by Porteous (1985), based on Schafer’s (1977) soundscape concept. It refers to the olfactory environment as perceived and understood by a person influenced by memories and past experiences, specific to its context (Xiao et al., 2018, p. 106). When considering living spaces and everyday environments, the quality of the olfactory environment is important both psychologically, e.g., subjective evaluation, and physiologically, e.g., stress recovery (Annerstedt et al., 2013; Hedblom et al., 2019). Thus, an examination of the smellscape may contribute towards our understanding of the relationship between perceptual processes such as odour annoyance as well as general wellbeing (Naddeo et al., 2013). Smells are closely associated with memories (Wilson and Stevenson, 2003; Herz, 2016). As the senses combine to influence our overall experience in everyday environments (Spence, 2020, p. 2) throughout our lives, memories contain cross-modal information that was concurrently encoded. Specifically, auditory, visual, and olfactory memories are interrelated. As olfactory perception exerts a large influence on the subjective evaluation of environments, we posit that when presented with either visual and/or auditory information, smells can be imagined to match this visual/auditory information, which would in turn exert an influence on the subjective evaluation of a particular environment.

## Smell Perception

Humans have several neurological systems to collect information about the environment (Spence, 2020, pp. 8–9). The olfactory system detects airborne semiochemicals (i.e., odours and scents) in particular in the orthonasal region at the front of the nose; the trigeminal system detects strong irritators (e.g., ammonia or chilli); and the vomeronasal (accessory) system detects chemicals in fluids, including pheromones (Haviland-Jones and Wilson, 2010; Lundström et al., 2011). The number of genes expressing odour detection through the nose constitutes one of the largest gene families in the genome (Buck and Axel, 1991, p. 183), perhaps up to 3% of the total genome which would make it second only to the immune system (Haviland-Jones and Wilson, 2010, p. 236). The human sense of smell compares rather favourably with that of dogs and rats (see McGann, 2017, for a review), and its discriminatory powers might be several magnitudes larger than what was previously believed (Bushdid et al., 2014).

The terminology pertaining to olfactory stimuli is inconsistent in the literature. Terms such as ‘odour’ and ‘smell’ sometimes appear to be used interchangeably (e.g., Haviland-Jones and Wilson, 2010; Bruce et al., 2015) and regardless of their valence; however, ‘malodour’ is specifically negative, while no such negation can be attached to the word ‘smell’. Likewise, Belgiorno et al. (2013) define ‘odour’ as an “organoleptic attribute [property of liquids, air, and other substances, that is] perceptible by the olfactory organ” (p. 15), and elsewhere appear to use ‘smell’ with the same meaning. Spence (2020) omits ‘odour’ in favour of ‘scent’, and in his review of design approaches to the olfactory environment appears to use ‘smell’ more often negatively, and ‘scent’ (or ‘fragrance’) in positively valenced contexts. Discussing olfactory imagery, Young (2020) consistently uses ‘smell’ when referring to the percept, and words such as ‘olfaction’ in the context of active sensing of the environment; the latter distinction is also made by Xiao et al. (2018).

Smells communicate. Smells have a strong effect on individuals, and may even induce emotional responses for unattended stimuli in a pre-conscious manner (Haviland-Jones and Wilson, 2010, pp. 237–238). Compared to visual and auditory stimuli, there has been much less of a consensus in quantifying olfactory stimuli for empirical analyses. Naddeo et al. (2013) proposed six parameters for characterising smells: by concentration, perceptibility threshold, intensity, diffusibility or volatility, quality, and hedonic tone. Of interest to the present study are intensity (perceived as strength), quality (or character), and hedonic tone (overall pleasantness or unpleasantness of a smell, and its resultant perceptual ‘acceptability’; cf. Dravnieks et al., 1984; Xiao et al., 2018).

The specific quality or character of a smell is often expressed via semantic descriptors, for example ‘fruity’ or ‘medical’ (Naddeo et al., 2013, p. 14). As with all sensory channels, olfactory sensation is mediated through individual factors, previous experience, and attention. Language embeds salient experiences and mediates between sensation and cognition, though a distinction needs to be made between different forms of cross-modal correspondences: what Deroy and Spence (2016) labelled statistical, structural, semantic, and hedonic (emotional) mediation mechanisms. If we accept the principle of the Lexical Hypothesis – that socially relevant characteristics of personality are encoded in natural language because this benefits social structures and individual survival (see John and Srivastava, 1999, for a review) – then salient odours in the environments need also to be communicated and understood within a linguistic group or culture for much the same reasons. This approach allowed the development of odour classification schemes, for example McGinley’s Odor Descriptors Wheel (McGinley and McGinley, 2002), which was employed in the present study.

People typically describe and categorise smells by perceived source (e.g., fishy, floral) but they also use words that describe the effect a smell has on them (e.g., nauseating, pleasant). Several categorisation studies have observed that people’s default mode of perception is ecological; that is, we tend to interpret sensation in terms of causes, as evidence of events and actions in the environment. For auditory perception, ecological conditioning over the lifetime lies behind the default mode of causal (or connotative) listening (Schaeffer, 1966; Chion and Gorbman, 2009; Tuuri and Eerola, 2012; see Lindborg, 2019, for a discussion). In this regard, olfaction appears to work much in the same way as auditory perception (e.g., Bruce et al., 2015; Deroy and Spence, 2016; Xiao et al., 2020) in that both the attributed external source and its subjective affect can be verbalised (Lindborg, 2016). As with causal identification of sound sources, an evaluation of smell sources follows immediately and automatically upon source identification (Waskul and Vannini, 2008; Xiao et al., 2020, p. 11) and probably regardless of whether the identification was correct or not (Herz, 2016). Dravnieks et al. (1984) determined the hedonic score (i.e., pleasantness) of 141 commonly encountered smells from ratings by 429 participants. Note, however, that people often cannot identify or use accurate descriptions to define smells or smell-sources, especially in a de-contextualised condition (Xiao et al., 2020), and that unconscious detection of smells most probably also contributes to the overall perception, though it might depend more on the trigeminal than the olfactory system proper (Jacquot et al., 2004, p. 51).

Congruence between sensory information channels leads to perceptual processing fluency which in turn is associated with positive evaluation (Reber et al., 2004; Spence, 2020, p. 2). It has been suggested that concurrent visual information can increase the ability of smells to arouse emotions, and that visuals without smells might evoke odour-related memories (Ehrlichman and Bastone, 1992). Gottfried and Dolan (2003) revealed neurological evidence for perceptual olfactory facilitation when semantically congruent visual and odour stimuli were presented. Castiello et al. (2006) investigated the cross-modal influence of smells on vision and motor activity. They presented people with a smell and then tasked them to grasp with their hand a virtual visual object. The imagined size of the smelly object influenced the size and shape of the hand as it was moved towards the visual object, showing that odours can cross-modally affect kinematics. Xiao et al. (2020, p. 14) wrote that “smellscapes are representations of individuals’ imaginations of places, triggered by smells in a space-time structure”. There is evidence that smells can trigger episodic memory recall (Herz, 2016) and that smells congruent with audiovisual displays can generate more positive response behaviour within a context of virtual tourism (Flavián et al., 2021). Might this cross-modal influence from smell to other senses be activated in reverse? Considering a theoretical framework for multi-modal mental imagery, Young (2020) posited that non-olfactory stimuli can trigger smell memories, which are projected as imagined smells.

Considering the above, we investigated if people can make meaningful smell associations when presented with purely audiovisual material. The present article discusses results from two recent studies of the smellscape at a complex multimodal environment, real and imagined. The first is a ‘sensory walk’ conducted onsite, with participants walking through seven locations while making observations. The observations from the first study functions as a reference or ground truth for the second study, which is a ‘virtual sensory walk’ conducted online, where the same locations were reproduced with audio and video recordings. Analysis of response data from the real environment provides a baseline against which ratings of the virtual environment were gauged (cf. Annerstedt et al., 2013). The online environments were represented in three conditions: audio-only, video-only, and audiovisual (i.e., movie). We were interested in determining the quality, strength, and hedonic tone of the smellscapes evoked and imagined (Naddeo et al., 2013).

## Materials and Methods

### Sensory Walk

Sensory walk and sensewalk are terms describing an activity whereby people move through a physical environment making observations with a research purpose. A soundwalk focuses on the acoustic environment and a smellwalk focuses on the olfactory environment. All are methods for onsite data collection that allow researchers to systematically investigate how people experience, understand, and utilise spaces (Henshaw et al., 2009, 2010; Bruce et al., 2015; Koseoglu, 2016; McLean, 2017; Xiao et al., 2020). The methodology originates in soundscape studies (Schafer, 1977) and has been broadened out to consider multimodal aspects of environments (Bruce et al., 2015, p. 100; Quercia et al., 2015). Carefully curated smellwalks can take the form of experience design (see Aggleton and Waskett, 1990) or as “walkalong interviews in different contextual spaces” (Xiao et al., 2020, pp. 15–16) that aim to generate an ecologically valid vocabulary for further semantic and cross-modal analysis. Porteous (1985, p. 360) suggested that while a soundscape consists of sound sources evidencing events, a smellscape should be understood as the sum total of numerous smell sources that each may connote cause and effect. While a soundmark is a sound that is intimately linked to a site and carries meaning for its community, a smellmark would correspondingly be a culturally highly important smell.

### Tiong Bahru Market

In order to study the perception of smells within a multimodal context, we conducted a sensory walk called “Incomplete City Walks: Coffee Shops and Hawker Centres” (Magiera, 2020) at Tiong Bahru Market in Singapore. Perhaps more than in other countries, open markets (aka ‘wet markets’) are socially important to Singaporeans because they are spaces for unmediated, non-political interactions (Mele et al., 2015). The history and significance of Tiong Bahru Market in this context was the main reason for choosing the site. Houses and shophouses appeared at the site of current-day Tiong Bahru Market around 1900. A nearby cemetery provided its name: ‘tiong’ (终) means “to die’ in Hokkien (the most prevalent language and culture until the 1980s) and the Malay word ‘bahru’ means ‘new’. By the 1930s roads, drains and culverts had been constructed, and one of the first public housing estates in Singapore was opened at Tiong Bahru in 1936. During this time, the area was also known as Mei Ren Wo, “den of beauties’, with musician-entertainers doubling as prostitutes referred to as ‘pipa girls’. It was generally considered an unseemly and unhygienic place. The marketplace roof was made up of palm leaves woven together, and the surrounding huts had thatched roofs (National Heritage Board, 2013). Tiong Bahru Market reopened in 2006 after thorough renovations over more than a decade (CPG Consultants, 2005). At present it sports a triangular-shaped architecture in three levels: the ground level has a wet market, shops selling vegetables, flowers, clothing, and hardware, and a green space; the second level has a large food court, a.k.a. a hawker centre; and the rooftop has a Carpark (Bravo, 2021).

### Locations

**FIGURE 1:**
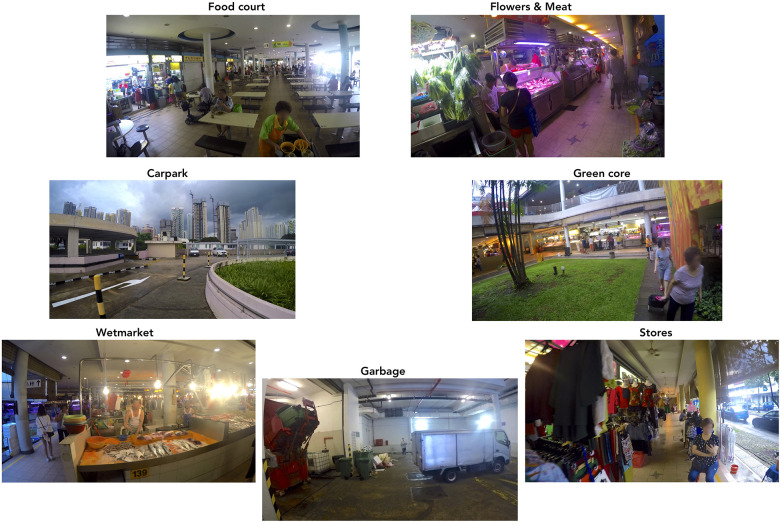
Photos of the seven chosen locations at Tiong Bahru Market.

**FIGURE 2:**
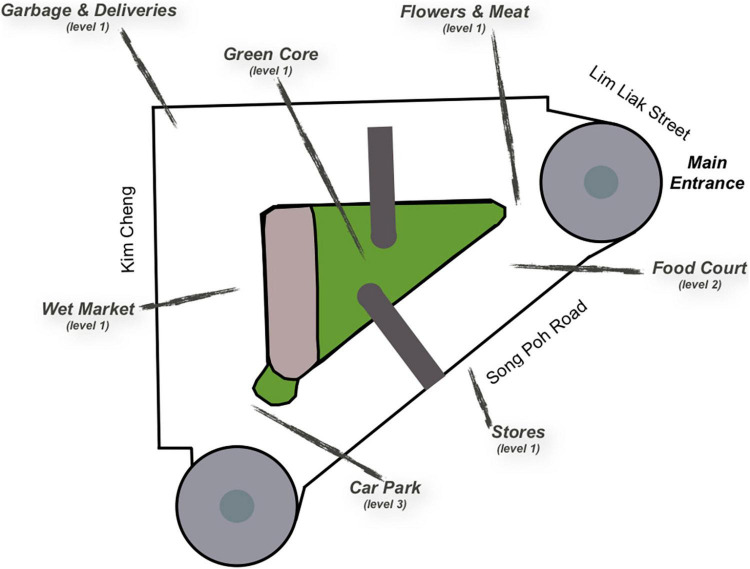
Overview of Tiong Bahru Market marking out the seven locations of the sensory walk.

Seven locations at Tiong Bahru Market were identified as having a strong identity in terms of smells and sounds. In the present text, they are labelled (in alphabetical order) Carpark, Flowers and Meat, Food Court, Garbage, Green Core, Stores, and Wetmarket. See photos in Figure 1 and map in Figure 2.

### Onsite Sensory Walk

#### Participants and Procedure

We posted a call on social media to participate in an early morning sensory walk at Tiong Bahru Market, saying *“In this structured sensory walk we explore a public space differently, outside of routines and habits. Moving slowly between locations affects our sense of architecture, spatiality, and time. Standing still and focussing on sound and scent increases our awareness of the physical environment. What do you hear? What do you smell? What do you experience?”*

Fifteen people volunteered. Their mean age was 36 years (SD = 12), in a range between 22 and 53; there were nine females and five males (and 1 ‘other, or prefer not to say’). As for present occupation, nine self-reported as artists, educators, or researchers, and two were students. They had mixed levels of previous experience with sensory walks or soundwalks: four had ‘never tried’, five had participated at a similar experience within the past month, and three had done so at an earlier point in time.

The participants gathered at 6.30 am, 3 December 2016, and were informed about the task and signed a consent form. None of the participants was smoking tobacco before or during the sensory walk. The group was divided into three groups of five, each led by the two authors and an assistant. The three groups of five completed the sensory walks among the seven selected locations of the market in a different, pseudo-random order. The whole walk took between 70 and 90 min that is, 10–14 min at each location. Moving from one location to the next took a few minutes (especially when walking stairs between levels. Upon reaching a new location, the group leader made sure the five participants stayed relatively close together and within the target area for around 7–8 min. Each participant carried a small support plate (A5 size) with the seven locations on separate pages arranged in the order of visit pre-planned for each of the three subgroups. They marked responses with a pencil while standing; the market did not have any benches to sit down on, except at the Food Court. At each location, the participants spent the first 2–3 min focussing on being still and taking in the environment, then 3–4 min on writing down their impressions, and finally, they were at ease for a few more minutes before the group leader signalled for the group to move on. The participants were admirably concentrated throughout the sensory walk, and a little after 8 am gathered for a debriefing over traditional breakfast.

#### Annotation Protocol

At each location, the protocol first required an evaluation of the quality of the sonic, visual, and olfactory aspects of the environment. Participants marked a response on a seven-step Likert scale anchored by “Very good” and “Very bad”, with “Neutral” in the middle. Secondly, they were required to describe “the most *faint* or *secretive* smell (or sound); the most *loud* or *dominant* smell (or sound); the most *beautiful* or *precious* smell (or sound); and the most *ugly* or *disgusting* smell (or sound)”. That is, they were free to annotate sources and events in either of the two modalities, in relation to the four given pairs of adjectives that had been set up as opposites. Third and last, participants were asked to describe, in a few words, their thoughts and feelings while being in the present environment. The above three responses or annotations are the ones of relevance to the present context; other parts of the protocol that related to soundscape will not be further discussed here. A sample page is included in Supplementary Data Sheet 1. It should be noted that asking participants to focus on smells (either real or imagined) might mask the full range of odour effects, because verbal explicitation focuses on learned semantic processes (see Haviland-Jones and Wilson, 2010, p. 239 for a discussion). Methods for onsite data collection were approved by the Institutional Review Board of Nanyang Technological University #IRB-2015-10-056.

The raw collected data from the two studies, anonymised, are available in Supplementary Data Sheet 2, 3. For convenience, a code script is given in Supplementary Data Sheet 4 to facilitate retrieval and pre-processing of the data.

### Online Virtual Smellwalk

#### Participants

Participants were recruited from Prolific.co and pre-screened for nationality (discussed below), age (minimum 18), and educational level (minimum completed Bachelor degree). Fifty-three valid responses with no duplicates were collected in March and April 2021. Each participant gave their informed consent and received a payment. All reported having no impairment of hearing or vision (wearing glasses/lenses was okay), being in a distraction-free environment during the survey, and using quality headphones (earbuds were discouraged).

Since the environment at Tiong Bahru Market is strongly characteristic of South-East Asia, we chose to recruit participants who may be culturally familiar (see Mele et al., 2014). We first recruited a batch of 25 Singaporean nationals, but upon a stagnation in signup rates, expanded our recruitment criteria to include a second batch of 26 participants from neighbouring Malaysia and Indonesia (that arguably share similar cultural attitudes towards wetmarkets; see Lim, 2004). It should be noted that Prolific.co is a United Kingdom-based company and that it mainly sources its pool of volunteers in Western countries. A minority of the participants were residing in their home country at the time of the survey; all were in countries that are indexed among the first 27 in UN’s list of Developed Countries, including Singapore, Malaysia, and Indonesia. For example, 25 participants were based in Great Britain, 8 in Germany, and 6 in Australia. Finally, two Hong Kong residents (not the authors) who had volunteered as beta-testers were included, bringing the total number of participants to 53.

Their median age was 27 years, in a range between 19 and 54; there were 36 females and 17 males. As for present occupation, 19 were employed (e.g., medicine, education, business) and 28 students (similar domains), while the remaining 6 self-reported as homemakers or unemployed. English was the home language for 33 participants, followed by Bahasa for 12 (spoken in Malaysia and Indonesia), while the remaining 8 reported Chinese (the given options included Mandarin, Cantonese, and Hokkien). Probably reflecting behaviours in their current place of residence rather than those of South-East Asia, participants reported infrequently visiting an open-air market (for shopping, eating/drinking, or buying things), with the most common response being “Once a month” followed by “Once a year”. As for ethnicity, 18 out of the 25 Singaporeans self-reported as Chinese, while on the other hand only 5 of the 28 other participants considered themselves Chinese (note that the second batch consisted of Malaysian and Indonesian nationals). The median time to complete the survey was 30 min, in a range between 14 and 62. They were paid according to the recommended Prolific hourly rate, which came out to be 6.25 GBP per person.

#### Materials

Video and sound materials for all stimuli used in the online study were captured at the same time that the onsite study was conducted, that is, in the early morning of 3 December 2016. The first author carried handheld recording equipment which did not detract from the role of (silently) leading the small group of five participants from location to location. Similarly, the Sound Pressure Level (SPL) meter was carried by the second author. In post-production, we edited a representative sequence of each location as a short movie of 90 s duration. Care was taken to create a naturalistic visual representation with an objective point of view, and to keep the sound levels proportional to what had been measured onsite with the SPL meter. We then created ‘audio only’ and ‘video only’ sets by replacing the original video with a medium-gray Gaussian blur and the original audio with low-level pink noise. This yielded 21 stimuli, i.e., seven scenes in three modes (referred to as audio, video, and movie). Note the importance of adding low-level yet audible noise to the ‘video only’ stimuli, since previous research has shown that a realistic visual feature without any sound at all can bias participant perceptions towards fear, and make them expect something dangerous to appear (Annerstedt et al., 2013).

#### Procedure

A survey was designed using QuestionPro^1^. The 21 stimuli were presented in two parts. First, an individually randomised part containing the seven Audio and the seven Video clips; then, an individually randomised part with the seven Movie clips. In this way the order effect of bimodal stimuli giving unwanted clues to their unimodal versions was avoided. For each stimulus, the participant was requested to go full-screen, start the movie (which could be greyish video with naturalistic audio, or video with low-level noise, or naturalistic audio and video together), and imagine that they themselves were actually in the present environment. The survey constituted an ‘onsite virtual sensory walk’, which we can compare with the onsite walk (functioning as ground truth). Participants were given three tasks to evaluate the audiovisual environment presented to them.

**FIGURE 3:**
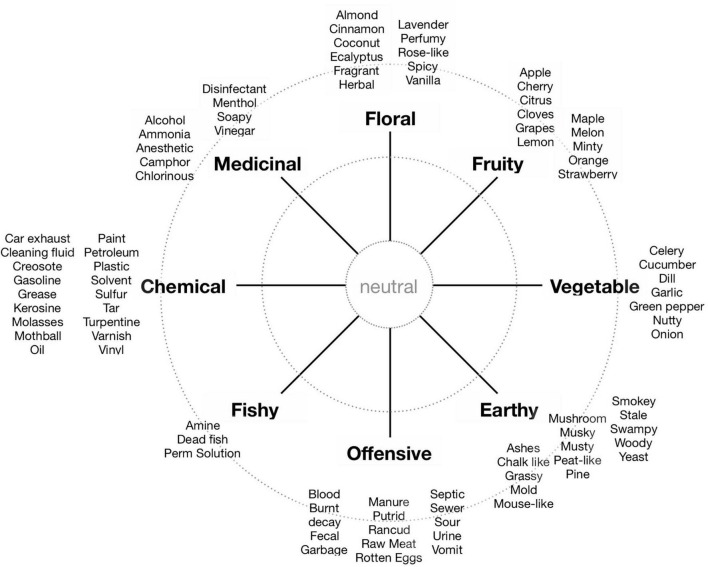
The ‘smellwheel’ used in the present study (adapted from McGinley and McGinley, 2002).

• The question “Overall, how pleasant is this environment?” was intentionally a broad, multimodal quality evaluation that did not specifically require them to imagine smells. They responded using a seven-step Likert scale anchored by “Very unpleasant” and “Very pleasant”, with “Neutral” in the middle.
• The instruction: “In your own words, describe the smells that you imagine”, with responses in the form of annotations of free associations, was intended to direct their attention specifically to the smells they might have imagined from audio-visual information.
• The Odor Descriptors Wheel (McGinley and McGinley, 2002) was displayed, in a slightly adapted layout (see Figure 3), and the participant was required to “Please click on the smell types that you imagine in this environment”. They could mark up to three smells by mouse-clicking (the average number of clicks per stimulus and participant was 2.6).

Note that the first two tasks provided two angles onto the phenomenon of how audiovisuals elicit smell imaginations, which we have suggested can explain the results in the statistical analysis further below. Note that no olfactory stimuli were presented to the participants, who only imagined the smells.

After completing all 21 stimuli participants self-reported demographic information (the group profile is reviewed in the “Participants” section above). Online data collection procedures were approved by the Ethics Committee of City University of Hong Kong, #13-2020-08-E.

## Results From Onsite and Online Studies

### Onsite: Real Smells

The results from the onsite study functions as a ‘ground truth’ against which the results from the online study can be interpreted. Therefore it is firstly of importance to explain how the onsite participants (*N* = 15) described their experience at the seven locations. The general experience of the environment was annotated by the onsite participants in free-form verbal descriptions. The locations had clearly different characteristics and we will discuss their olfactory aspects by citing representative annotations verbatim.

• Carpark: this location was described in mostly visual terms, e.g., “View – sense of open space, aerial, above”.
• Flowers and Meat: this is a mixed-market area that evoked spatial and olfactory annotations, e.g., “meat – stronger smell”, “The smell is totally different in flower to meat area”, and “Flowers do not smell as strong as I usually experience @Flower hawkers” highlighting an activity-related expectation.
• Food court: this is a typical Singaporean-style ‘hawker stalls’ area serving various kinds of local food. It engaged all sensory modalities, though as one participant wrote: “here, smells of foods cooking dominate other senses. Must be ready for breakfast!”, while another highlighted the continuous, open-air character of Tiong Bahru Market: “Olfactory, continuous with car park [on Level 3], but not L1 market”.
• Garbage: as discussed in the section about environmental quality ratings, it was generally considered unpleasant. However, the smells were not necessarily as strong or bad as some appear to have expected: “It was quite monotonous in the sense that I didn’t feel much for that place [Garbage] despite attempting to discover its sounds and smells”. Meanwhile, another person tried to “focus specifically to LISTEN [and this] makes it a more pleasant experience, and I didn’t notice the smell at all as I normally (probably) would”. Lastly, one person remarked that it was “interesting how smells move from place to place, as does sound”, highlighting the characteristic spatial pervasiveness and temporal continuity of smell – these characteristics are stronger for smell than for sound, and clearly more so than for light.
• Green Core: the small park is at the connecting centre between other locations. Smells and sounds reach it from all directions and cause blurred sensations. One participant characterised it as “…a hot pot of noise. Everything can be heard but cannot be heard clearly at any one time. Nothing makes sense”, and the same may have been the case for smells, e.g., “Smell – dense; Busy; Sound – interesting”. For at least one participant, their expectation of a ‘park smell’ was not met: “Wish i could smell the wet grass over the raw meat”, perhaps indicating that the smell of raw meat was still dominating even though the row of butchers was some 20 m away.
• Stores: this location is a row of small shops along a street. Here, participants mostly noted sonic elements, such as the background music played in the small shops. One participant reacted strongly to the “SMELL of incense shop at [the] end of [the] row [which] gave me an immediate headache and this created a strong negative association with what had been super-pleasant. The changing music of the stalls, as I walked along, was calming and gave joy”.
• Wetmarket: this is an area selling fish and seafood. Annotations revealed marked expectations about the odours, but also that “fish variety of colour doesn’t correspond to smell”, perhaps indicating that smells tended to blend together. One person annotated a specific ‘precious’ source (see below) as “Delicate smell of dried scallops, cuttlefish”, but to most of the participants in this study the Wetmarket smells were simply generically ‘fishy’ and did not distinguish themselves. An intriguing observation about social communication habits was that “People seem to talk more with their fishmongers than [with their] butchers”. Previous research has noted that the observed activities lead people to expect and imagine particular sounds and smells in certain areas (e.g., Bruce et al., 2015, p. 8).

#### Environmental Quality

**FIGURE 4:**
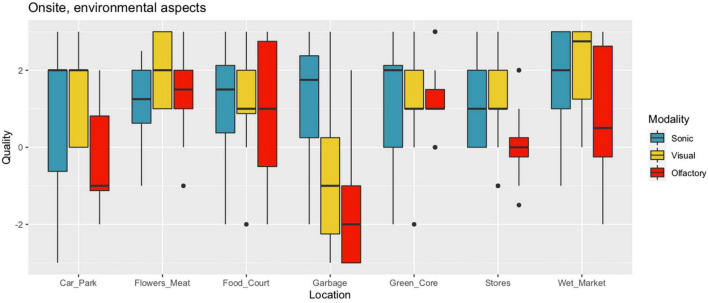
Boxplots of onsite ratings (*N* = 15) of three aspects of environmental quality for seven locations. Quality was rated on a seven-step Likert scale; see the text for details.

The onsite participants (*N* = 15) rated the perceived olfactory quality of the environment, as well as the visual and sonic aspects of the environment. Multivariate analysis of variance (MANOVA) revealed that quality ratings (i.e., olfactory, visual, and sonic) differed between the seven selected areas of the open market [Pillai’s trace (6 df) = 0.677, *F*(18,222) = 3.60, *p* = 0.000003]. This significant result allowed us to follow up with a two-way within-participants ANOVA, taking Quality as the dependent variable and Location and Modality as the independent variables. Each of the independent variables was strongly related to the dependent variable [Location: *F*(6) = 8.56, *p* = 0.00000002; Modality: *F*(2) = 10.1, *p* = 0.00006]. *Post hoc* analysis with Tukey’s Honest Significant Difference test showed that Olfactory ratings at the Garbage location were much lower than those at the six other locations, corresponding to as much as two or three steps on the seven-step Likert scale. With the Garbage location removed from the data (keeping the remaining six locations), Olfactory quality ratings were still significantly lower than Sonic and Visual quality ratings, as indicated by Mann–Whitney’s rank-based test on paired ratings (*p* = 0.031 for Olfactory vs. Sonic; *p* = 0.0006 for Olfactory vs. Visual). The differences, respectively, corresponded to 0.53 and 0.86 steps on the rating scales, with medium effect sizes (Cohen’s *d* = 0.33 and 0.55 SD). The difference in quality between Sonic and Visual aspects of the environment was not significant (*p* = 0.88) across seven locations, though when Garbage was removed, Visual quality was marginally higher than Sonic across the six other locations (0.03 scale steps, *p* = 0.041) with a small effect size (*d* = 0.21) (see Table 1, Col. 1–3, and Figure 4).

**TABLE 1:** Mean ratings of environmental quality and hedonic tone in the two studies.

	Onsite, environmental quality			Online, environmental quality			Onsite, hedonic	Online, hedonic tone		
	Col. 1	Col. 2	Col. 3	Col. 4	Col. 5	Col. 6	Col. 7	Col. 8	Col. 9	Col. 10

	Sonic	Visual	Olfactory	Audio	Video	Movie	Olfactory	Audio	Video	Movie
Carpark	0.88	1.54	−0.31	0.00	0.26	0.09	−0.52	−0.73	−0.08	−0.69
Flowers and Meat	1.14	2.00	1.42	0.00	0.08	−0.45	0.80	−0.43	−0.03	1.81
Food Court	1.12	1.21	1.05	0.28	0.30	0.83	−0.03	0.35	0.16	1.08
Garbage	1.21	−0.83	−1.54	−1.08	−2.23	−1.75	−2.48	−2.43	−4.87	−5.10
Green Core	1.21	1.23	1.27	−0.09	0.42	0.26	0.30	−0.01	0.72	0.36
Stores	1.27	1.19	0.12	−0.11	0.49	0.34	0.00	−0.81	−0.81	−0.50
Wetmarket	1.71	2.11	0.71	0.04	−0.43	−1.30	−1.44	−0.29	−0.41	−3.03

*In Col. 1–3 (onsite) and Col. 4–6 (online), values are means of ratings on seven-point Likert scales (numerically limited between −3 and 3), averaged across 15 participants in the onsite data, and 53 participants in the online data. Values in Col. 7 (onsite, hedonic value, olfactory) were obtained by matching all the onsite annotations of smells in free-form verbal descriptions with smell descriptors and corresponding hedonic scores in from previously published research (Dravnieks et al., 1984). Scores were summed for each location and averaged across the 15 participants. Values in Col. 8–10 were obtained by the same method, from free-form annotations in the online data. They were summed for each location and modality (audio, video, movie), and averaged across the 53 participants in the online study.*

It is not immediately obvious why the olfactory environment received lower quality ratings than both the visual and the sonic aspects. Note that the participants at the onsite sensory walk had been tasked to ‘seek out’ the smells, and this might have predisposed them to be more critical to smells. However, it is also conceivable that the bias did not originate in the participants’ minds but that it reflects something true about the environment at Tiong Bahru Market. For example, the market might simply not be successful in taking care of and promoting the grand variety of its smellscape in a positive way; rather, it is left hanging, as it were. The major renovation (CPG Consultants, 2005), and habits of the vendors, might have paid less than adequate attention to the design of the olfactory environment, which therefore lags behind the quality of visual or sonic aspects of the market. For example, a current website promoting the market (Bravo, 2021) does not highlight its smellscape. Previous research noted that the odours of fish are generally expected and accepted as part of the experience of a wetmarket, regardless of what someone might otherwise think of the smell of raw fish (Bruce et al., 2015, p. 7). In fact, for Tiong Bahru Market, the smell of raw fish is a smellmark (Porteous, 1985). A suitable presentation in marketing material might prepare visitors (e.g., tourists) and thereby enhance the experiences of Tiong Bahru market as a whole, benefitting the larger economy (Henshaw et al., 2016). Attention to the smellscape as part of the intangible heritage has a potential to benefit ‘virtual tourism’, as shown by Flavián et al. (2021).

#### Smell Sources

**FIGURE 5:**
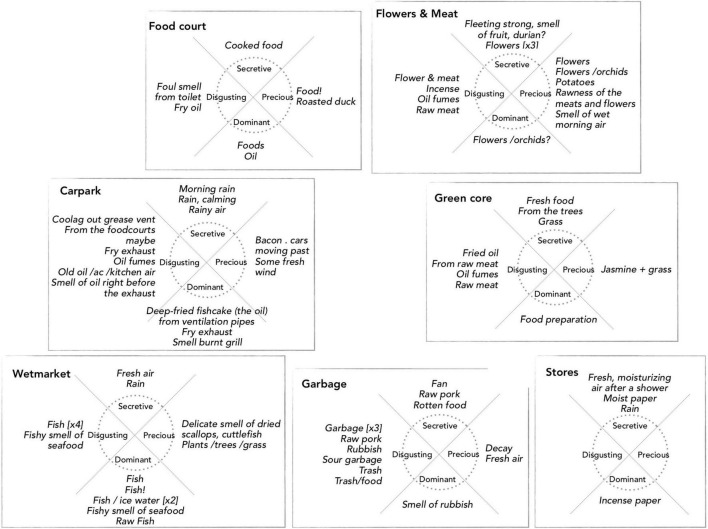
Onsite smell source annotations (*N* = 15) for seven locations; see the text for details.

The onsite participants annotated smell sources at each of the seven locations and indicated whether they considered them to be *precious* (or beautiful), *secretive* (or faint), *disgusting* (or ugly), or *dominant* (or loud). We chose the four adjectives because they can be paired two by two, to define bipolar, orthogonal dimensions labelled precious – disgusting and secretive – dominant. Thus, in this analysis, precious smells oppose disgusting smells, and secretive smells oppose dominant smells. The approach allowed arranging smell sources in a circumplex, one for each location, as shown in Figure 5. Note that the circumplex spanned by the four adjectives is a simple yet adequate approximation to the more complex smellwheel (Figure 3), where the left-right and low-high directions are intrinsically congruent with the dimensions in a standard Valence-Arousal circumplex. That is, high valence corresponds to *more precious*; low valence to *more disgusting*; high arousal to *more dominant*; and low arousal to *more secretive*. However, be aware that the smellwheel does not directly yield information about the valence of individual smells, and can only indirectly be used to indicate the arousal potential (e.g., intensity) of sources by considering the distance between the ‘neutral’ centre to a distinct smell type (more about this below). In Figure 5 the seven locations have been placed according to the main types in the smellwheel (compare also with photos in Figure 1). This graphical arrangement supports our interpretation of the locations’ general environmental characteristics, and facilitates a comparison with the online responses.

#### Hedonic Tone

The smell source annotations (in terms of precious – disgusting and secretive – dominant) allow us to infer which locations were more liked or more exciting. To estimate the hedonic tone (pleasantness) for each location, we matched the annotated smells against the list by Dravnieks et al. (1984), which contains 141 commonly encountered smells with hedonic tone rated on a nine-step Likert scale by 429 participants. The listed scores range from −3.75 for “Cadaverous (dead animal)” to 3.53 for “Bakery (fresh bread)”. Nearly all of our participants’ descriptions could be matched with items in the list. Notable unmatched annotations related to the smells of humidity after rain, fresh air, and wind, all of which likely to have been positively valenced for the onsite participants. It might be debatable to what extent ‘fresh air’ has a smell of its own, but one should take note of the fact that such usage of semantic labels by laymen is systematic (see also the corresponding analysis of the online data). In the present calculation of hedonic score, these annotations had to be omitted. Summing up within each location yielded the values listed in Table 1, Col. 7.

### Online: Imagined Smells

#### Environmental Quality

**FIGURE 6:**
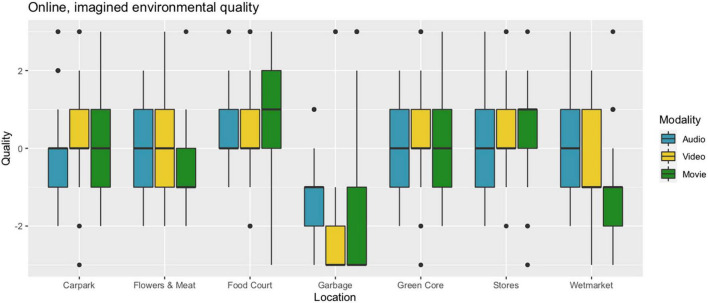
Boxplots of ratings (*N* = 53) of environmental quality in seven locations, as imagined when presented in three different audiovisual conditions. Quality was rated on a seven-step Likert scale; see the text for details.

In the online study, participants (*N* = 53) rated the imagined environmental quality in terms of pleasantness for each of the 21 stimuli, that is, seven locations and three modalities (audio, video, and movie; see Procedures section). We conducted a two-way within-participants ANOVA with Quality as the dependent variable, and Location and Modality as independent variables. It revealed a strongly significant relation between Quality and Location [*F*(6) = 61.3, *p* < 2e-16], but not with Modality [*F*(2) = 1.632, *p* = 0.20]. The fact that there were no systematic differences in environmental ratings between presentation modalities (i.e., conditions) suggests that participants were on the whole equally able to judge environmental quality from audio-only and video-only stimuli as they were with movie-stimuli. However, further analysis revealed a strongly significant interaction between Location and Modality [*F*(12) = 7.56, *p* < 2e-13], which led us to conduct a series of *post hoc* analyses with Tukey’s Honest Significant Difference test. Similarly to the onsite study, it was found that the Garbage location was lower rated than all the six others, corresponding to between 1.1 and 2.2 steps on the seven-step Likert scale, with a large-sized effect of 1.37 SD (Hedges’ g with correction for different group sizes). Furthermore, the Wetmarket was lower rated than the five remaining locations, corresponding to between 0.44 and 1.1 scale steps, with a medium-sized effect of 0.62 SD (Hedges’ *g*, corrected). Lastly, the Food Court was rated 0.6 scale steps higher than Flowers and Meat, with a medium effect of 0.53 SD (Cohen’s *d*) (see Table 1, Col. 4–6, and Figure 6). The largely corresponding results between the two studies in terms of perceived environmental quality will be probed further below.

#### Smell Sources

The smellwheel response interface has ‘neutral’ at the centre and eight main types of smell evenly distributed in cardinal directions: Vegetable, Fruity, Floral, Medicinal, Chemical, Fishy, Offensive, Earthy. Each of the eight types is exemplified with several subtypes (see Figure 3). Participants could mark with a mouse click up to three smells that they imagined in the present environment (audio-only, video-only, or movie). They were asked to “click at the centre (inner circle) if they imagined the smellscape as neutral, and further out (outer circle) if they imagined that smells would be strong”.

Participants marked on average 2.6 smells per stimulus; there were 2907 clicks in all. They tended to be placed close to the eight spokes of the smellwheel or at the centre. Within the inner ‘neutral’ circle, there were 13.9% of the clicks; between inner and mid circles, 54.8% of clicks; between mid and outer circles, 21.8%, and outside the outer circle, 9.5%. The analysis proceeded by interpreting smellwheel responses in terms of smell type (discrete, eight kinds) and smell strength (continuous). In the present analysis, the click’s distance from the centre is taken as a proxy for the strength (perceived intensity) of the identified type of smell, its numerical value relative to the radius of the outer circle. For example, a smell strength value of 0.5 means that the participant clicked half-way between the centre and the outer circle. We observed a correlation between the order of clicks and their distance from the centre. The mean distance for the first clicks that participants made was 0.514, for the second 0.505, and for the third 0.468. Analytic tests comparing the distributions of clicks, i.e., 1st versus 2nd and 2nd versus 3rd, were significant in both cases (*p* < 0.0001, Mann–Whitney’s rank-based test on paired samples), however the effect sizes were small (0.08 and 0.15 SD, respectively). This order effect can be explained by assuming that the smell that participants imagined to be strongest was also the first one that they clicked on, and for ensuing smells the imagination was less certain, or weaker. Moreover, the number of clicks could also be a useful measure for smell source strength, as shown by the fact that distance from centre and click count (within locations, modalities, and smell types) were highly correlated (distance correlation dcor = 0.59, *p* < 0.0002 for 5,000 bootstrap replications).

##### Type

**FIGURE 7:**
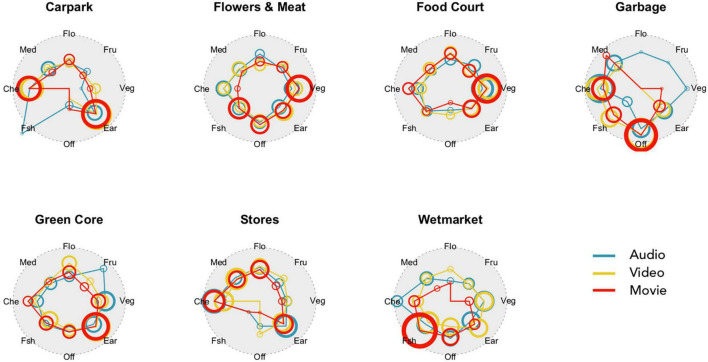
Graphical illustration of smell types (qualities) and intensities in seven locations and in three modalities. Smell types are abbreviated for clarity: Veg, vegetable; Fru, fruity; Flo, floral; Med, medicinal; Che, chemical; Fsh, fishy; Off, offensive; Ear, earthy. Colours refer to modalities, i.e., stimuli conditions. For each smell type and modality, the coloured ring is centred on the mean distance of clicks, and its thickness and size are proportional to the number of clicks made.

Figure 7 illustrates graphically (in the style of Belgiorno et al., 2013, p. 15) the relative intensities of McGinley’s eight smell types in the seven locations and three modalities. From inspection, it is clear that one or two smell types were imagined to be dominant at each of the locations, and the three modalities were largely congruent. At the Carpark, the dominant types were Chemical and Earthy; at Flowers and Meat and the Food Court, Vegetable smells dominated; at Garbage, the Offensive smells were dominant; at the Green Core mini-park, Earthy smell types were prevalent; at the Stores, Earthy and Chemical types; and finally at the Wetmarket, the Fishy smells were dominant. This is very similar to the smells detected by the onsite study participants, see Figure 5. Note also that the differences between modalities in the online virtual smellwalk were not striking. In the Audio-only condition, the types were more evenly distributed, but even at the Garbage location, the participants imagined largely the same kind of smell types as they did when video was present.

##### Strength

**FIGURE 8:**
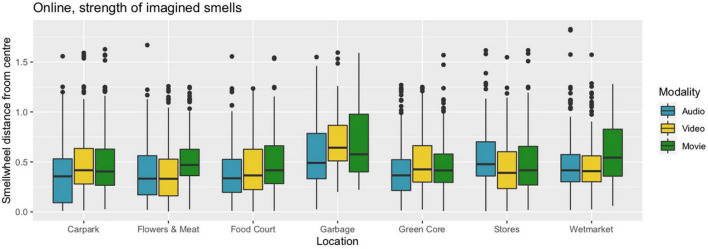
Imagined smell intensity (proxied by mouse-click distance from smellwheel centre; see the text for details) in seven locations and three audiovisual modalities.

Unsurprisingly, analysis of variance revealed significant differences of imagined smell strength between locations and modalities. This is illustrated in Figure 8. For locations, Tukey’s Honest Significance test showed that smell strength was higher at the Garbage location than at any of the other six. The difference was on average 0.17 (range 0.12…0.21; recall that strength takes a value from 0 to about 1), with an overall medium-sized effect of 0.54 SD (Hedges’ g with correction for different group sizes). It was also significantly higher at the Wetmarket than at Flowers and Meat or Food Court; the difference was about 0.08 for both, with an overall effect size of 0.27 SD. For modalities, the same test showed that imagined smell strength was higher in the Movie condition than in the two unimodal conditions. The difference between Movie and Audio was 0.08 (Cohen’s *d* = 0.27 SD), and between Movie and Video it was 0.05 (*d* = 0.17 SD).

#### Hedonic Tone

The onsite participants were asked to imagine the smells in the represented environment (audio, video, or movie), and describe them using their own words. There were 1113 free-form responses (7 × 3 × 53), each consisting of four words on average. They were pre-processed by removing non-letter symbols (e.g., question marks, citations, parentheses, trailing spaces), transcribing to lowercase, and merging grammatical variations, e.g., singular and plural. There were a total of 2561 individual words, out of which 344 were unique. The 33 most common were: food (6.0%), fish (4.0%), meat (3.9%), grass, neutral (2.5%), raw (2.0%), rain (1.9%), chemical, car (1.8%), exhaust, fresh, vegetables, air (1.6%), earthy, garbage (1.4%), clothes (1.2%), incense, market, musty, smoke, wet (1.1%), damp (1.0%), sweat, cooked, oil, dust, restaurant, humid, trash, humidity, new, rubbish, blood (0.7%). The frequency counts for each of the three modalities (audio, video, and movie) are listed in Table 2.

**TABLE 2:** Most common descriptors for annotations of imagined smells (*N* = 53) at seven locations in three modes of presentation.

	Audio	Video	Movie
*Carpark*	Neutral (9.4%), car (4.7%), chemical, rain, Air conditioning (3.5%), factory (3.5%)	Grass (14.0%), rain (13.0%), air (8.1%), exhaust (5.9%), car (5.2%), fresh, wet (3.7%), petrol (3.0%), after (2.2%), concrete, dust, open (2.2%)	Grass (15.0%), exhaust (9.8%), car (9.1%), rain, air (4.2%), earthy (3.5%), petrol, after (2.8%), dust (2.1%), fresh, pollution, smoke (2.1%)

*Flowers and Meat*	Food (11.0%), neutral, fish (4.5%), cooking (3.4%), meat (3.4%)	Food (9.4%), neutral (8.3%), coffee (3.1%), cooked, dust, people, sweat (3.1%)	Meat (19.0%), vegetables (18.0%), raw (10.0%), fish (7.4%), grass (3.7%), market, blood (2.9%), fresh, fruit, earthy (2.2%), food, wetmarket (2.2%)

*Food Court*	Food (20.0%), restaurant (6.9%), cooked (5.2%), neutral, tea (3.4%), air (2.6%), people (2.6%)	Food (18.0%), crowd (5.4%), restaurant, neutral (4.5%), cooked (3.6%), people (2.7%), sweat (2.7%)	Food (31.0%), cooked (4.6%), oil, garlic (3.8%), oily, cooking (3.1%), hawker (2.3%)

*Garbage*	Chemical (7.7%), water (4.6%), metal (3.8%), metallic, factory (3.1%), wood, carwash (2.3%), dirty, garbage, machinery, oil, smell, soap, trash (2.3%)	Garbage (13.0%), rubbish (7.1%), trash, rotten (6.2%), pungent (4.4%), putrid, bad (2.7%), chemical, rancid, sour (2.7%)	Garbage (12.0%), trash (6.8%), food (5.3%), putrid (4.5%), rotten, rubbish (3.8%), pungent (3.0%), sewer, fish (2.3%), offensive, rancid, rotting, sour, wet (2.3%)

*Green Core*	Meat (9.9%), food (9.0%), neutral (6.3%), raw (4.5%), restaurant (3.6%), smoke (2.7%)	Grass (21.0%), fresh (8.7%), meat (7.1%), fish (6.3%), earthy (4.0%), wet, air (2.4%), market, rain, raw, vegetables (2.4%)	Grass (19.0%), meat (9.5%), earthy (6.3%), raw, fish (4.8%), fresh, air (4.0%), food (3.2%), damp (2.4%), greenery, humid, market, wet (2.4%)

*Stores*	Car (7.9%), exhaust (7.1%), incense, smoke, fumes (3.6%), traffic, medicinal (2.1%), medicine, neutral, street, sweat (2.1%)	Clothes (11.0%), incense (6.9%), new, musty (5.5%), rain, chemical (4.8%), fabric, dust (2.8%), medicinal, air (2.1%), clothing, earthy, floral, humid (2.1%)	Clothes (11.0%), incense (8.1%), new (6.1%), chemical (5.4%), car (4.7%), musty, exhaust (4.1%), fabric (3.4%), floral, plastic (2.7%), Chinese (2.0%), medicinal, shop, smoke (2.0%)

*Wetmarket*	Meat (12.0%), food (7.9%), fish (4.0%), market, raw, sweat (3.2%), vegetables, blood (2.4%), fruit, hawker, neutral, people (2.4%)	Meat (14.0%), fish (10.0%), food (9.3%), market (5.1%), raw, sweat (4.2%), vegetables, butcher (2.5%), cooked, dust, wetmarket (2.5%)	Fish (45.0%), raw (5.8%), meat (4.9%), damp (2.9%), dead, humid, market (2.9%)

*The listed descriptors are those that occurred three times or more in each of the 21 stimuli.*

**FIGURE 9:**
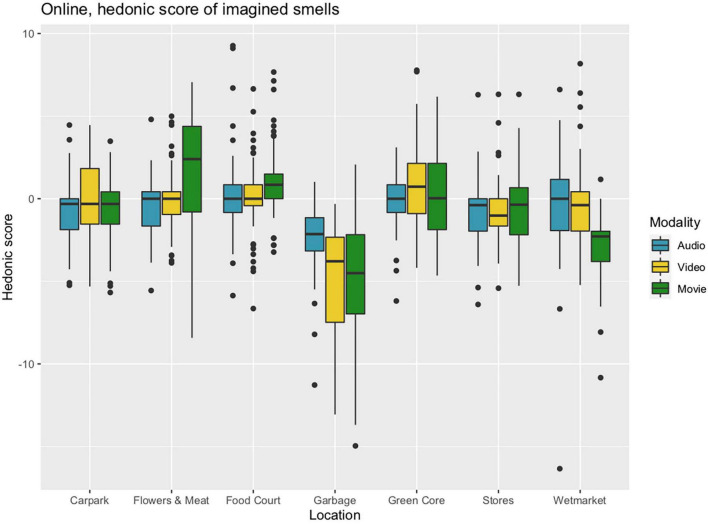
Boxplots of cumulative hedonic scores for seven locations and three modalities, estimated from free-form descriptions of imagined smells by raters (*N* = 53) matched against previous data (Dravnieks et al., 1984).

A cumulative score for hedonic tone for each location and modality was calculated from the descriptive words, in a similar way as for the onsite free-form annotations. We matched the 344 unique words from our participants with Dravnieks’ list (Dravnieks et al., 1984). For 27% of the unique words a perfect match was available; for 22% a close match was found (e.g., iron → Metallic); for 30% an acceptable match was made by considering the word in the context of its sentence [e.g., car → Gasoline, solvent, or food → Seasoning (for meat)]. For 20% of the words no match could be made (e.g., air, clothes, house). This process yielded a hedonic score for each participant’s free-form annotations of smell sources. Scores were accumulated across participants to give an estimate for the hedonic tone of each of the seven locations and three modalities. The distributions are illustrated in Figure 9.

Analysis of variance (ANOVA) revealed that there were differences between the locations. *Post hoc* analysis with Tukey’s Honest Significant Difference test showed that the Garbage location was lower rated lower than all the six others, corresponding to between 2.9 and 4.7 units (Dravnieks’ scale values, accumulated within each participant’s free-form response), with a large-sized overall effect of 1.47 SD (Hedges’ g with correction for different group sizes). Furthermore, when Garbage was removed from the data, the Wetmarket location was lower rated than the five remaining, corresponding to between 0.54 and 1.8 units, with a medium-sized overall effect of 0.50 SD (Hedges’ g, corrected). Tukey’s test also revealed that Carpark had lower hedonic tone than Food Court and Flowers and Meat, and that Stores was lower than Flowers and Meat, Food Court, and Green Core, though these differences will not be discussed further. Note that there were no systematic differences in hedonic tone between the three conditions of stimulus presentation.

As was already noted in the analysis of onsite data, some online responses that were frequent (and therefore probably important) could not be satisfactorily matched to Dravnieks’ list. For example, the matchings with food-related or rain-related descriptions were only acceptable, and no matching could be made for the relatively frequent descriptions involving [fresh] air or [new] clothes. Furthermore, only 69 of the 141 items were used at all; more than half were not relevant to the present study. This suggests that the overlap is not large between the smells (both real and imagined) perceived at a South-East Asian outdoor market and the smells in Dravnieks’ study from almost 40 years ago in Illinois, United States. Future research might pursue a cross-cultural approach to smellscape perception and the categorisation of smell sources.

### Comparison and Summary of Results

Finally we analyse the results from the two studies together and compare them in terms of environmental quality, hedonic tone, and smell sources. The onsite study represents the ‘ground truth’, in the sense that the olfactory environment was physically present to the participants even if they might not have been fully able to identify or label the smell sources. In the online study no smell variations existed physically, so all differences in annotations and ratings of smells were imaginary, and evoked by the audiovisual stimuli. Hence, it is of interest to compare results from the two, for those variables that are comparable. In what follows, we report results using Canonical Correlation Analysis (CCA) to compare ratings of environmental quality, and estimates of hedonic tone of the smellscape as constituted by smell sources, respectively. For these multivariate measures results from the two studies are available in numeric form (see Table 1). CCA is a dimensionality reduction technique that takes two datasets – matrices with an arbitrary number of columns (variables) – and finds the projection that accounts for the most of the covariance between the two (Thompson, 2000; for an application, see e.g., Eerola and Vuoskoski, 2011). Lastly, we make a summary qualitative comparison based on free-form annotations.

#### Environmental Quality

A comparison between Figures 4 and 6 suggests that an association exists between the two studies, in terms of ratings of environmental quality. We ask the Reader to bear in mind that the sonic, visual, and olfactory aspects of the real, multimodal environment and rated during the sensory walk, do not directly correspond to the audio, video, and movie representation modalities that were presented online as a ‘virtual sensory walk’. Moreover, in the online situation, participants were asked to make an overall evaluation of how pleasant the environment was imagined to be; they were not specifically required to imagine the olfactory environment. This, as we shall demonstrate, becomes important for how to interpret the results.

First, we use CCA to measure the overall association between the means of onsite overall quality ratings in three environmental aspects and the means of online pleasantness ratings in three presentation modalities (Col. 1–3 vs. Col. 4–6 in Table 1). The observed correlation between the first canonical variates from the two matrices is 0.99912. A *p*-value can be simulated by creating a distribution of correlations between two random matrices of the same sizes (both 7 × 3), over 50,000 repetitions. Using this bootstrap method, the simulated p-value is 0.107; this means that a spurious correlation as high as the one observed occurs 10.7% of the time. Thereby the data do not support inferring that the two situations are comparable in terms of the ratings for environmental quality. However, we can exclude the onsite olfactory ratings and calculate the measure of association between onsite sonic and visual aspects with the three audiovisual online conditions (Col. 1–2 vs. Col. 4–6 in Table 1). The first canonical correlation component is then 0.99869. The bootstrap distribution in this case is created on random matrices of different sizes (2 × 7 and 3 × 7), producing a simulated *p*-value of 0.0074. This is highly significant, and means that a spurious correlation as high as the one observed occurs only 0.7% of the time. Moreover, similarly testing the association between onsite olfactory aspects on its own with the three audiovisual online conditions (Col. 3 vs. Col. 4–6 in Table 1), yields a canonical correlation of 0.82494. The bootstrap distribution created on random matrices (1 × 7 and 3 × 7) yields a p-value of 0.28, which is clearly non-significant. These results show that while information about the quality of the sonic and visual environment is transferable from an onsite, real situation to an online, virtual situation, this is not the case for olfactory information. In our study, the online task did not specifically require participants to imagine the smellscape, and the onsite information about the olfactory environment was not spontaneously evoked and reconstituted through the audiovisual information alone.

#### Hedonic Tone

We run the same analysis between means of onsite hedonic scores and means of online hedonic scores in three presentation modalities (Col. 7 vs. Col. 8–10 in Table 1). Note that this comparison does not involve data from McGinley’s smell wheel, used in the online study, but only the free-form annotations which had been gathered in exactly the same way in both studies, onsite and online. The scores for hedonic tone were estimated via Dravniek’s list (Dravnieks et al., 1984). The observed first canonical correlation is 0.98486. The bootstrap distribution in this case is created on random matrices (1 × 7 and 3 × 7), producing a simulated p-value of 0.0086. This is highly significant, and means that a spurious correlation as high as the one observed occurs only 0.86% of the time. In terms of hedonic tone, an association between the two studies is supported by data. Nevertheless, the critical Reader will want to take into account that the estimates for both sets of data were based on Dravnieks’ study, which may or may not be ideal for the smells in the present situation.

#### Smell Sources

For smell source types and strengths, we interpret the onsite and online results with a qualitative approach without attempting to make strong conclusions. The real, onsite smell sources were distilled from free-form verbal annotations of source categories (Figure 5), while the imagined, online smell sources were those most frequently indicated by the smellwheel responses (Table 2).

• Carpark: Across onsite and online situations, descriptions typically refer to grass, cars, car exhaust, and rain. Note the mention of air conditioning in the audio-only condition (noticeable in the recording), through which wafts of smells from the food court on the level below came through.
• Flowers and Meat: The onsite descriptions highlight the mix of smells from flowers, vegetables, and raw meat. Meanwhile, online descriptions do not pick up on the flowers, and emphasise food-related smells, also in the audio-only condition.
• Food Court: In both situations descriptions of food smells abound. Onsite participants also identified foul smells that were not imagined by online participants. In the latter case, mentions of people were fairly common in audio and video conditions.
• Garbage: Here, descriptions onsite and in online video and movie conditions were dominated by words such as trash, pungent, and rotten foods. In the online audio-only condition, the more common words were about chemicals, water, metal, and machinery, and participants appear to have imagined a factory or carwash rather than a garbage handling area.
• Green Core: Onsite descriptions mix food smells and grass, which were also highlighted in the online descriptions in video and movie conditions. The presence of smells from grass, trees, and soil was not imagined in the audio-only condition.
• Stores: The smell of incense paper is annotated as dominant onsite. This was echoed in the online descriptions in all three conditions, which also imagine smells from car [exhaust]. The audio condition did not evoke the smell of clothes, which are clearly visible in the video (and movie) and are the most frequently described.
• Wetmarket: The smell of fish is clearly dominant onsite, and it is imagined in all three online conditions, although the audio-only condition evoked more general terms such as meat and food more frequently than the specific fish association.

In summary, smell source descriptions were in fairly high agreement between onsite and each of the three online conditions for the Carpark, Food Court, Stores, and Wetmarket locations. Notably, the audio-only condition in the online study failed to evoke significant smell imaginations for the Green Core and Stores locations, and most pertinently, the Garbage location did not contain sounds that triggered smell associations in this direction.

## Discussion

The olfactory system (in the brain) is closely connected to the limbic system, involving neural regions like the hippocampus, amygdala, and thalamus (Hedblom et al., 2019). Consequently, olfactory perception is also closely tied to emotional and stress processing, and smell-related autobiographical memories with associated emotional encodings are also retrieved with stronger activation of the amygdala, beyond non-emotional memories or emotional memories associated with other modalities (Gottfried et al., 2004; Herz, 2016). Consistently, perceived smells that are not actively attended to are also able to evoke emotional responses in individuals (Haviland-Jones and Wilson, 2010, p. 237). Smells can be incorporated into individuals’ schema for places (Tse et al., 2007; Henshaw et al., 2009), with strong connotations with affective and emotional associations. However, despite these emotional linkages, certain behavioural fight-or-flight decisions are often not concluded solely based on olfactory perception. Instead, smells appear to trigger a search for contextual meaning, or confirmation of the odour source, through other (visual or auditory) modalities (particularly for fear-related odours; Haviland-Jones and Wilson, 2010, p. 242), possibly in a bid to match or form new associations from long-term memory. If so, this suggests that cross-modal information is important to olfactory perception. This forms an angle to interpret our current results: we would expect to see systematic differences when people are asked to imagine smells when exposed to either sound- or visuals-only stimuli, or audiovisual (i.e., movie stimuli). Indeed, the highly significant association in terms of hedonic tone (pleasantness) between the online and onsite situations in our data suggests that when online participants were specifically required to imagine smells from audiovisual information, they were successful. They could approximate the types of onsite smell sources and their ‘actual’ perceived hedonic tone. Imagined smells may thus be a mechanism towards the emotional evaluation of audiovisual environments. By contrast, the analytical results in this study show that ratings of the quality of the olfactory environment (immediately available to onsite participants) did not translate to ratings of environmental quality from audiovisual information only (with no olfactory stimuli present) at least when online participants were not required to imagine smells from audiovisual information.

We may consider the difference between perception and cognition more broadly, and how it might apply in the case of the present study. What does it mean to say that someone has the smell of a fish ‘in the nose’, or that she has a mental representation of fish smells ‘in the brain’? Zach et al. (2018) conducted a study on olfactory, auditory, and visual volitional memory recall of French Fries among a group of mainly teenagers. They found that on-demand olfactory recall was at least to some extent possible for a majority of their subjects (73.2%, 71.0%, and 77.4% in their three groups, respectively). Levels were similar to those for auditory recall but much lower than those for visual recall. However, Zach also states that olfactory recall was harder to do for their subjects, perhaps because smells might take longer time to evoke by volition than sounds or sights. On the other hand, Young (2020) points out that imagination is a broader concept than memory recall: “olfactory imagery seems limited when only considered as the capacity to voluntarily self-generate an experience of olfactory quality in the absence of sensory stimulation. However, a more liberal conception including any experience of olfactory quality in the absence of sensory stimulation widely expands the instances of olfactory imagery by including dream states, hallucinations, autobiographical odour memories, and olfactory memories” (Young, 2020, pp. 3305–3306). In the subjective, free-form annotations in our present work, the evidence of type and strength of olfactory recall – imagining smell on demand and in the absence of stimulation by actual odours – is always going to be reported with different levels of vividness, both because olfactory imagination can be expected to vary greatly across the population, but also because of individual factors unrelated to olfaction (e.g., language abilities, attentiveness). Future research might attempt to validate self-evoked smell percepts through behavioural, physiological, and other external measurements. This reiterates the difficulty of spontaneously imagining smells to match audiovisual environments, since individuals often have to draw on their own familiar (culturally rooted) experiences to do so (Bruce et al., 2015). Referring to fear-odours, (Haviland-Jones and Wilson, 2010, p. 242) noted that “sensing an odour without having a visual or auditory match leads to a slow, cautious search for a match rather than the rapid fear-flight response”. This led the authors to propose that “there must be an ongoing, not-quite-conscious match-mismatch system that is correlating bits of information across the sensory systems. It is actively searching for existing associations and making new associations” (Haviland-Jones and Wilson, 2010, p. 245). If such a mechanism exists, we would expect to see systematic differences when people are asked to imagine smells when exposed to either sound- or visuals-only stimuli, or audiovisual (i.e., movie stimuli).

To a certain extent, the significant relationship identified between onsite and online participants in hedonic tone could be facilitated by a shared cultural background (similar schema towards the market). However, in real-world settings, one cannot always control the cultural backgrounds of viewers to a particular video. Given that olfactory perception has a stronger impact on stress reduction and other health-related paradigms (Annerstedt et al., 2013; Hedblom et al., 2019), this may lead to a cultural imbalance in the subjective evaluation of environments (such as through movies or online videos) that present only audio-visual information. Individuals may form incomplete or biased judgements about a location or environment of another (unfamiliar) culture, if presented solely with audiovisual information (e.g., a trailer to an exotic travel destination). This may in turn have implications on tourism strategies in general for a city or country (Henshaw et al., 2016; Spence, 2020, p. 8). The recent study by Flavián et al. (2021) provides evidence that congruent smell presentation as part of a multimodal VR display acts to increase positive behavioural responses in the context of ‘virtual tourism’. We refer the interested Reader to their article which also contains an excellent review of recent development in olfactory-extended virtual reality (Flavián et al., 2021).

This issue of schema, memory and association in imagining smells is evident in the discrepancy of descriptions between locations in the study. For example, some locations, such as the Carpark, that were less subject to cross cultural specificity, appeared to have more qualitative consistency between onsite descriptions and online imaginations. By contrast, the onsite descriptions of the Wetmarket frequently featured fish-related smells, as opposed to the imagined meat-related smells that were prevalent in imagined online descriptions. One explanation for this discrepancy could be in the direction of attention. The auditory/visual stimuli forces the viewer to follow a top-down specified path of attention for smell-imaginations, which can be influenced by one’s upbringing and culture (Masuda et al., 2020). On the other hand, given the source-confirmation drive often associated with olfactory perception, onsite participants could have first relied on olfactory perception as a preliminary input, before searching for the source through audiovisual confirmation in a bottom-up approach.

## Limitations and Future Work

The current findings are limited in that no olfactory stimuli were presented during the online study. The article by Flavián et al. (2021) is exemplary in its design of an experiment with olfactory conditions when a small number of distinct smells are tested. It is inspiring for our further work on the relationship between real smellscapes (‘in the nose’) and imagined smellscapes (‘in the head’), which will be needed in order to test the findings we have reported here. A further limitation is the substantial time gap between conducting the onsite study at Tiong Bahru market, and the online study. We had hoped to carry out a confirmatory second onsite study, in the form of a sensory walk, but unfortunately the COVID-19 restrictions during the summer of 2021 made it impossible. Outlets at the market were closed or working at minimal capacity; e.g., no customers were allowed to sit at food court tables, and no gatherings of people were allowed anywhere. Waiting for restrictions in Singapore to be lifted would delay publication of the present text. Instead, we are turning our energies towards a follow-up project in Hong Kong (where the first author is currently based). It will include a survey of several wet markets, with collection of audio-visual recordings and olfactory measurements together with observations made during sensory walks. An interesting option for an onsite study might be to partially inhibit multisensorial perception, for example by blindfolding some participants, having others wear hearing muffs, and others again with nose clips. Experiments in the laboratory will include a suitable way of replicating the relevant smells of wet markets, for example by presenting olfactory stimuli corresponding to the main parts of the smellwheel (McGinley and McGinley, 2002) by using odour compounds in ceramic vessels or on perfume sticks.

Future work will also pay close attention to different kinds of memory. For example, when asked to imagine smells on demand, the memory one recalls might be semantic (“knowing”; common knowledge shared by others) rather than episodic (“remembering”; a person’s autobiographical olfactory experiences). For example, when asked the question “In your own words, describe the smells that you imagine”, a participant who has never been to a wet market might still be able to write down words like “fishy”, “seafood”, and so forth, simply using knowledge they learned in school. In our present online study we enrolled participants from Singapore and neighbouring countries in South-East Asia, allowing the assumption of them having personal experience with Asian-style open air markets. However, it is a limitation of our data elicitation method that it did not make a clear distinction between episodic and semantic memories. In future online studies, it might be of interest to include a ‘no external stimulus’ condition where participants are required, for example, to recall the last time they were in the specified environment and to describe the smells they imagine based on their lived experience.

## Conclusion

Analysing perceptions of the built environments, Jean-Paul Thibaud wrote that “urban ambiances are created and experienced as a product of different, sometimes unique, blends of sights, sounds, smells, textures, tastes and thermal conditions” (Thibaud, 1995, p. 204). Smell represents an important dimension of our understanding and design of the everyday environment. In particular, the olfactory environment offers benefits for well-being and restoration both in architecture (Spence, 2020, pp. 6–7) and in the context of calm, green, natural places (Hedblom et al., 2019). To be of optimal usefulness for urban design, research on smellscape perception should consider the olfactory environment in relation to individual factors (Lindborg and Friberg, 2016; Xiao et al., 2018). The urban smellscape indeed forms part of our intangible heritage (Lenzi et al., 2021). It is valuable as culture, and at the same time, it also represents a marketing opportunity (Henshaw et al., 2016), especially for urban servicescapes that contain a multitude of sensorial information (Bruce et al., 2015, p. 6; Lindborg, 2015).

Recent interdisciplinary initiatives show the way towards a ‘World Smellscape Project’ based on the model of soundscape research (Schafer, 1977). Smellscape measurements and descriptions made by ‘nose-witnesses’ (Porteous, 1985, p. 360) constitute an archive of experiential descriptions of various smells around the globe. Notably, the Odeuropa project^2^, which is involved in intangible cultural heritage and everyday culture, aligns with our present work. Meanwhile, D-Noses^3^ focuses on odour pollution control, and GoodCityLife^4^ works at improving the urban living environment through parallel smart-city mapping projects. The citizen science approach also doubles up as a resource for the historical archival of locations and places as they develop over time. Through this paper, we present a humble contribution to this larger discourse through a documentation of the smellscape of an area of significance in Singapore’s history, while examining cross-modal linkages between olfactory perception and audio-visual perception.

## Data Availability Statement

The raw data supporting the conclusions of this article will be made available by the authors, without undue reservation.

## Ethics Statement

The studies involving human participants were reviewed and approved by Institutional Review Board of Nanyang Technological University #IRB-2015-10-056, and Ethics Committee of City University of Hong Kong #13-2020-08-E. The patients/participants provided their written informed consent to participate in this study.

## Author Contributions

PL conceived the study and conducted the analysis. KL and PL collected the onsite response data and PL collected the online data. Both authors collaborated on writing and reviewing all parts of the manuscript. Both authors contributed to the article and approved the submitted version.

## Conflict of Interest

The authors declare that the research was conducted in the absence of any commercial or financial relationships that could be construed as a potential conflict of interest.

## Publisher’s Note

All claims expressed in this article are solely those of the authors and do not necessarily represent those of their affiliated organizations, or those of the publisher, the editors and the reviewers. Any product that may be evaluated in this article, or claim that may be made by its manufacturer, is not guaranteed or endorsed by the publisher.

## Footnotes

1
www.questionpro.com

2
https://odeuropa.eu/

3
https://dnoses.eu/

4
https://goodcitylife.org/

## Supplementary Material

Supplementary Data Sheet 1 Sample page of the protocol used in the onsite sensory walk.

Supplementary Data Sheet 2 Collected raw data from Study 1, onsite (CSV).

Supplementary Data Sheet 3 Collected raw data from Study 2, online (CSV).

Supplementary Data Sheet 4 Code example (R script) for retrieving and pre-processing of the raw data.
