# Supplementary material for: Real and Imagined Smellscapes
Source: Front Psychol. 2021 Dec 24;12:718172. doi: 10.3389/fpsyg.2021.718172 (PMC8740324; doi:10.3389/fpsyg.2021.718172)
Supplement: Supplementary Data Sheet 1 — Sample page of the protocol used in the onsite sensory walk. [file Data_Sheet_1.pdf]

# Food Court (level 2)

**Environment** - **Overall,** ***what is your evaluation of different aspects of the environment?***

| | Very good | Neutral | Very bad |
|---|---|---|---|
| Sonic | | | |
| Visual | | | |
| Olfactory | | | |

**Sources** - ***To what extent do you hear the following five types of sounds?***

| | Not at all | Average | Dominates |
|---|---|---|---|
| Nature, birds, wind | | | |
| Music | | | |
| Traffic, cars, airplanes | | | |
| Fans, machines, noises | | | |
| Cooking, cutting, tableware | | | |
| Conversation, individuals | | | |
| Crowds of people | | | |

**Description** - ***In the present environment, in a few words, describe the…***

| | |
|---|---|
| most FAINT or SECRETIVE<br>Sound ☐ or Smell ☐? | |
| most LOUD or DOMINANT<br>Sound ☐ or Smell ☐? | |
| most BEAUTIFUL or PRECIOUS<br>Sound ☐ or Smell ☐? | |
| most UGLY or DISGUSTING<br>Sound ☐ or Smell ☐? | |

**Soundscape** - ***To what extent do you agree with the adjectives below on how you experience the present sonic environment?***

| | Not at all | Neutral | Completely |
|---|---|---|---|
| Pleasant | | | |
| Chaotic | | | |
| Vibrant | | | |
| Uneventful | | | |
| Calm | | | |
| Annoying | | | |
| Eventful | | | |
| Monotonous | | | |

**Experience** - ***In a few words, please describe your thoughts and feelings while being in this environment.***
