# Supplementary material for: Real and Imagined Smellscapes
Source: Front Psychol. 2021 Dec 24;12:718172. doi: 10.3389/fpsyg.2021.718172 (PMC8740324; doi:10.3389/fpsyg.2021.718172)
Supplement: Supplementary Data Sheet 4 — Code example (R script) for retrieving and pre-processing of the raw data. [file Data_Sheet_4.pdf]

```r
## ======================================== SMELLSCAPES: REAL AND IMAGINED
## scripts to read raw data and start preprocessing
##
## if you want full code used in the analysis, please contact me using the email below!
## mosst of the analysis code is OK documented, other parts are less so.
## sometimes custom wrappers & convenience functions etc were used
##
## PerMagnus Lindborg 2021
## pm.lindborg@cityu.edu.hk


# ======================================== LIBRARIES
if (!"gsheet" %in% installed.packages()) (install.packages("gsheet"))
library(gsheet)
if (!"readr" %in% installed.packages()) (install.packages("readr"))
library(readr)
if (!"openxlsx" %in% installed.packages()) (install.packages("openxlsx"))
library(openxlsx)
if (!"data.table" %in% installed.packages()) (install.packages("data.table"))
library(data.table)
if (!"psych" %in% installed.packages()) (install.packages("psych"))
library(psych)

getwd()
list.files()

# ========================================================================================== ONLINE` PREP DATA
onsitedata <- read.csv("2-Smellscape-Onsite-rawdata-anon.csv")
summary(onsitedata)

mycols <- colnames(onsitedata)
pcpinfo <- mycols[1:11]
overall <- mycols[14:16]
sources.scal <- mycols[17:23]
sources.descr <- mycols[24:31]
quals.protocol <- mycols[32:39]

locations <- sort(as.character(unique(onsitedata[,"Location"])))

str(onsitedata)
onsitedata$ID <- factor(onsitedata$ID)
onsitedata$Gender <- factor(onsitedata$Gender)
onsitedata$Lasttime <- factor(onsitedata$Lasttime)
onsitedata$Location <- factor(onsitedata$Location)
onsitedata$Faint_type <- factor(onsitedata$Faint_type)
onsitedata$Loud_type <- factor(onsitedata$Loud_type)
onsitedata$Beautiful_type <- factor(onsitedata$Beautiful_type)
onsitedata$Ugly_type <- factor(onsitedata$Ugly_type)

colnames(onsitedata)

# ========================================================================================== ONSITE` PREP DATA
raw_online <- read.xlsx("3-Smellscape-Online-RawData-anon.xlsx")
ncol(raw_online)
## note that empty columns are dropped
colnames(raw_online)
raw_online[1:8]
stim <- 21
ix <- 1:8 + 8*(stim-1)
raw_online[ix]

##
rawstims <- strsplit("TB1bV | TB5bV | TB3bV | TB6bA | TB1bA | TB3bA | TB2aV | TB4bV | TB2aA | TB5bA | TB7bV | TB4bA | TB6bV | TB7bA | bimodal | TB7bM | TB6bM |
 TB4bM | TB5bM | TB3bM | TB2aM | TB1bM", " | ")[[1]]
rawstims[seq(1, 43, 2)][-15] ## remove the 'bimodal' marker, doublecheck with QuestionPro ordering

block_labs <- c(sort(c(sprintf("%iA", 1:7), sprintf("%iV", 1:7))), sprintf("%iM", 1:7))
good_colnames <- c("Descr", "x1", "y1", "x2", "y2", "x3" , "y3", "Overall")
coll <- NULL
for (b in 1:21) {
    coll <- c(coll, sprintf("%s_%s", block_labs[b], good_colnames))
    }
coll
length(coll)
##
colnames(raw_online)[1:168] ## responses
colnames(raw_online)[169:180] ## participant data

### ---------------------------------------------------------------------- some checks
onlinedata <- raw_online
colnames(onlinedata)[1:168] <- rep(good_colnames, 21)
colnames(onlinedata)[1:168] <- coll
str(onlinedata)

colnames(onlinedata)

nrow(onlinedata) ## wide format, not going to use it
ncol(onlinedata)

## doublechecking
onlinedata["4M_Descr"]
mean(onlinedata[, "4V_Overall"]) ## low  == unpleasant
mean(onlinedata[, "5V_Overall"]) ## high  == pleasant
```
